# Physiological and Antioxidant Responses of Rough Lemon (*Citrus jambhiri* L.) Rootstocks to Foliar-Applied Green-Synthesized ZnO Nanoparticles Under Drought Stress

**DOI:** 10.3390/plants15193050

**Published:** 2026-10-06

**Authors:** Monaza Tehseen, Yi Hou, Tariq Aziz Khan, Muntaha Munir, Xuelian Zang, Zhenzhen Cao, Luna Hu, Chunhua Zhou, Lun Wang

**Affiliations:** 1 College of Horticulture and Landscape Architecture, Yangzhou University, Yangzhou 225009, China; monazatehseen@gmail.com (M.T.); houyi0311@163.com (Y.H.); tariqazizz987@gmail.com (T.A.K.); 17663972975@163.com (X.Z.); mz120241605@stu.yzu.edu.cn (Z.C.); mz120231492@stu.yzu.edu.cn (L.H.); 2 Department of Horticulture, Bahauddin Zakaria University, Multan 60800, Pakistan; 3 Department of Botany, University of the Punjab, Lahore 54590, Pakistan; muntahamunir03@gmail.com; 4 Jiangsu Key Laboratory for Genetic Improvement and Efficient Utilization of Horticultural Crop, Nanjing 225009, China

**Keywords:** antioxidant defense, citrus rootstocks, drought stress, green synthesis, nanobiotechnology, photosynthesis, rough lemon seedlings, zinc oxide nanoparticles

## Abstract

Drought stress affects citrus growth and physiological responses by impairing photosynthesis and antioxidant homeostasis. In this study, we produced green-synthesized zinc oxide nanoparticles (ZnO NPs) using leaves from *Calotropis gigantea* L. We assessed their effects on drought-stressed rough lemon (*Citrus jambhiri* L.) seedlings. UV–Visible spectroscopy, X-ray diffraction (XRD), scanning electron microscopy (SEM), and dynamic light scattering (DLS) confirmed the formation of hexagonal wurtzite ZnO nanoparticles with predominantly spherical morphology and nanoscale particle size. The synthetic material had a particle size of about 10 nm. We conducted a pot experiment under well-watered and drought conditions, withholding irrigation for 15 days and applying foliar ZnO-NP at 0, 50, 100, 150, and 200 ppm. Drought caused decreased plant growth, biomass, gas exchange, PSII performance, and chlorophyll relative content. ZnO application reduced these effects, and 150 ppm produced the strongest responses in plant height, number of leaves, biomass, SPAD, and PSII-related traits. Additionally, under drought conditions, 100–150 ppm ZnO also positively affected photosynthetic performance and antioxidant responses such as SOD, CAT, POD, TPC, and DPPH activity. Overall, green-synthesized ZnO NPs, especially at 100–150 ppm, enhanced the physiological attributes and antioxidant defense of rough lemon rootstocks under water-deficit conditions. These results indicate that they could be a nano-enabled method for citrus drought management.

## 1. Introduction

Nanotechnology is a new technique for reducing drought-induced oxidative damage because its unique physicochemical properties help deliver nutrients to plants and increase drought tolerance. Among the available nanoparticle types, zinc oxide (ZnO) nanoparticles are of particular interest because zinc is an essential micronutrient and plays a role in membrane stability, enzyme activation, chlorophyll synthesis, and metabolism during photosynthesis [1]. Nano-scale fertilizers have distinct physicochemical characteristics, including a high surface-area-to-volume ratio, which enables slow nutrient release and increased leaf uptake compared to large-size chemical fertilizers [2,3].

The application of ZnO nanoparticles in agriculture is attributed to their unique physicochemical properties, which enhance nutrient use efficiency and enable controlled nutrient release compared with conventional fertilizers [4]. These include the size, shape, crystallinity, surface charge, concentration, and formulation of the nanoparticles, which also dictate how they are taken up, moved, and interact with plant tissues [5]. These traits also play a role in abiotic stress responses, such as increased photosynthetic efficiency, antioxidant activity, and nutrient balance in plants [6]. Hence, their optimization is crucial for achieving the positive effects of ZnO nanoparticles in an agricultural context with minimal potential phytotoxicity [7].

The biological activity of ZnO nanoparticles depends not only on their chemical composition but also on their physicochemical properties (such as particle size, crystal structure and morphology, stability, and surface charge), which affect uptake, transport, and interactions with plant tissues. The use of plant extracts as reducing agents for the eco-friendly and economically viable synthesis of metal nanoparticles has been proposed as an alternative to conventional chemical and physical synthesis methods, which are often carried out under harsh conditions with toxic reducing agents and hazardous chemicals [8]. The effectiveness of green-synthesized, foliar-applied ZnO nanoparticles derived from *C. gigantea* in alleviating drought-induced morphological, physiological, and biochemical disturbances in rough lemon (*Citrus jambhiri* L.) rootstocks is particularly suitable for green synthesis because its leaves are rich in bioflavonoids, enzymes, phenolics, and alkaloids, which can act as natural reducing and stabilizing agents during ZnO nanoparticle synthesis [9,10].

The use of biologically synthesized nanoparticles to improve abiotic stress tolerance has been evaluated in a few perennial fruit crops, but only to a limited extent. Previous studies have shown that chemically synthesized metal oxide nanoparticles can reduce malondialdehyde (MDA) accumulation and improve antioxidant enzyme activities in plants such as maize [11], tomato [12], and soybean [13] under drought stress. However, limited information is available regarding the physiological and biochemical responses of citrus rootstocks to green-synthesized ZnO nanoparticles under drought stress. Specifically, the growth, photosynthetic performance, and antioxidant defense mechanisms of rough lemon (*Citrus jambhiri* L.) in response to biosynthesized ZnO nanoparticles have not been thoroughly studied [14,15]. This knowledge gap is significant because the establishment and sensitivity of citrus tree roots during the nursery stage critically affect the productivity, adaptability, and sustainability of commercial citrus orchards under water-limiting conditions [16].

Citrus is one of the largest and most important fruit crops planted worldwide, but its yield is highly susceptible to abiotic stresses, especially drought [17]. Water deficit is considered a major inhibitor of plant growth in arid and semi-arid climates, disrupting gas exchange, slowing photosynthesis, and eventually reducing crop performance [18,19]. The presence of ROS induces severe oxidative stress, resulting in the degradation of membrane lipids, nucleic acids, and the photosynthetic apparatus [20,21].

We hypothesized that foliar application of ZnO NPs at 100–150 ppm would provide an effective concentration range for enhancing drought tolerance, as recent evidence shows that moderate ZnO-nanoparticle application can activate antioxidant defenses, reduce oxidative damage, and protect photosynthetic cellular structures under drought stress. The selected range therefore represented an evidence-supported effective dose while providing a moderate concentration for physiological protection [22,23].

The species was selected as a medicinal herb because of its high concentration of secondary metabolites, which can act as environmentally friendly reducing and stabilizing agents during nanoparticle formation [24]. Therefore, the present study evaluated the effectiveness of green-synthesized, foliar-applied ZnO nanoparticles derived from *C. gigantea* in alleviating drought-induced morphological, physiological, and biochemical disturbances in rough lemon (*Citrus jambhiri* L.) rootstocks. In particular, the study examined the effects of ZnO nanoparticle concentrations (0, 50, 100, 150, and 200 ppm) on plant growth, biomass production, gas-exchange characteristics, photosynthetic performance, relative chlorophyll content, and antioxidant responses under well-watered and drought-stress conditions. The study also sought to find the optimum concentration of ZnO nanoparticles for enhancing drought resistance in rough lemon rootstocks [25,26].

## 2. Results

### 2.1. Characterization of Green-Synthesized ZnO Nanoparticles

**Figure 1:**
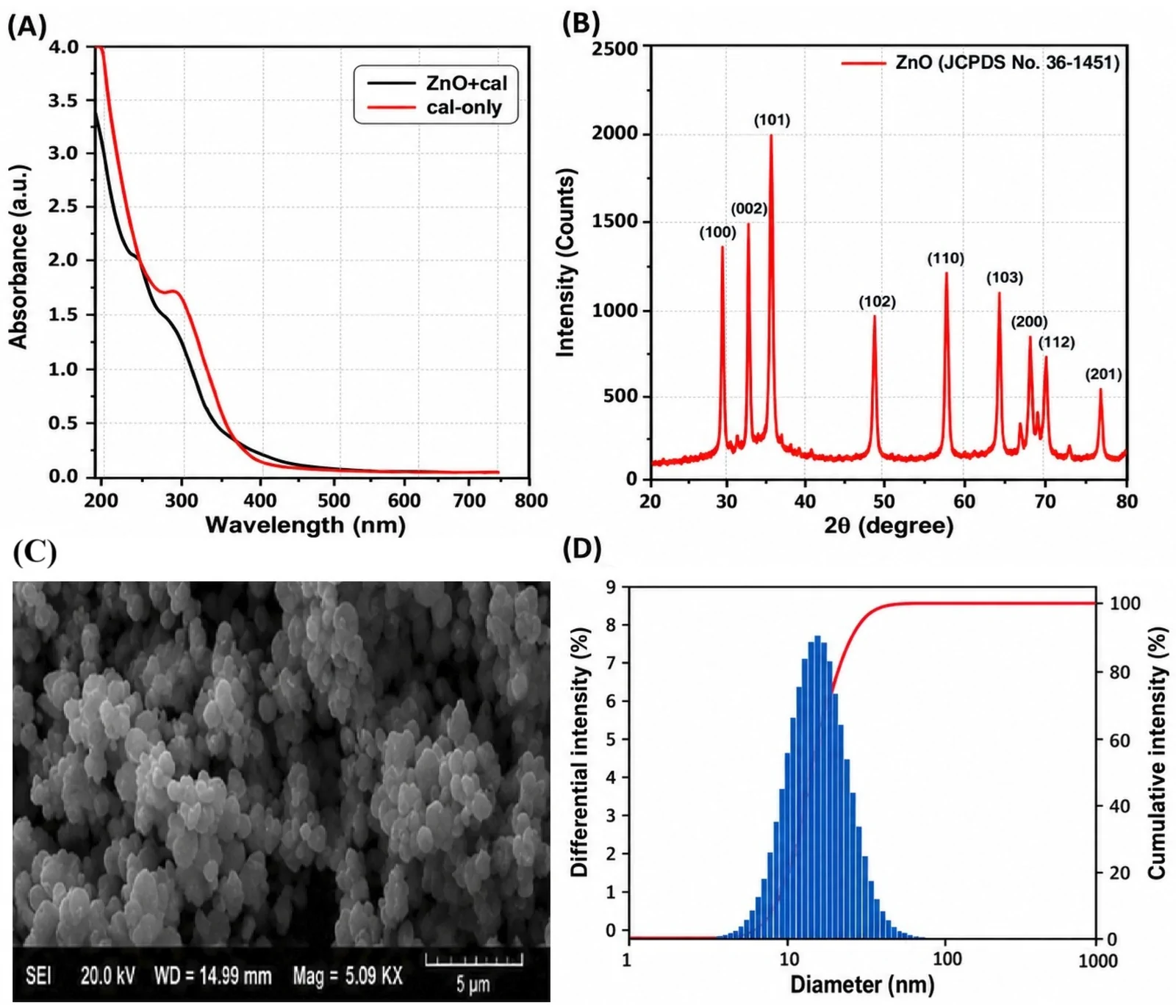
Characterization of green-synthesized ZnO nanoparticles using (**A**) UV–Visible spectroscopy, (**B**) X-ray diffraction (XRD), (**C**) scanning electron microscopy (SEM), and (**D**) dynamic light scattering (DLS) analysis. Morphological features, spectra, diffraction patterns, and differential intensity confirmed the synthesized ZnO nanoparticles and revealed particle formation and structural features.

UV–Vis spectroscopy (Figure 1A), structural (Figure 1B), morphological studies (Figure 1C), and Dynamic Light Scattering (DLS) analysis (Figure 1D) revealed the formation of ZnO NPs via the *C. gigantea* leaf-extract-mediated route, with an average particle size of approximately 10 nm. This nanoscale dimension is an important physicochemical characteristic because smaller particle dimensions generally provide a higher specific area, which can influence surface reactivity and interactions with biological targets. UV–Vis spectroscopy (Shimadzu Corporation, Kyoto, Japan) (Figure 1A) showed a change in the absorption envelope in the ultraviolet region for the ZnO-containing sample compared with the extract-only control. Because the leaf extract absorbs in the same region, the ZnO-containing sample’s absorption envelope indicated band-edge absorption typical of ZnO and suggested particle formation, supporting the optical evidence. X-ray diffractometer (D8 Advance, Bruker AXS GmbH, Karlsruhe, Germany) (Figure 1B) confirmed the crystalline phase of the product in the range of 20–80°, which showed diffraction peaks that correspond to (100), (002), (101), (102), (110), (103), (200), (112), and (201) planes of hex. wurtzite ZnO (JCPDS No. 36-1451). The sharp, well-defined reflections, with the most intense being the (101) reflection, indicate a well-crystallized wurtzite phase, and no prominent additional reflections of secondary crystalline phases were detected. The morphology of the particulates and their state of aggregation were determined by the corresponding scanning electron microscope (JSM-6490LA, JEOL Ltd., Tokyo, Japan) (Figure 1C; 5.09 kX magnification; scale bar 5 μm); the micrographs in this case indicate the predominant shape (rounded to quasi-spherical) and aggregation (present in the form of aggregates) of the particulates rather than a quantitative size distribution at the magnification used. We used a Zetasizer Nano ZS90 (Malvern Panalytical Ltd., Malvern, UK) to determine the average particle size and size distribution of biosynthesized ZnO nanoparticles (Figure 1D). The average hydrodynamic diameter of 10 nm indicated that the synthesized green ZnO nanoparticles were nanoscale. The particle-size distribution also showed that the synthesized ZnO NPs were mostly nanoscale. Based on the UV–Vis, SEM, XRD, and DLS data, the extract-mediated reaction generated ZnO nanoparticles with a hexagonal wurtzite structure and a spherical-like, agglomerated particulate morphology.

### 2.2. Assessment of Plant Height and Number of Leaves per Plant

**Figure 2:**
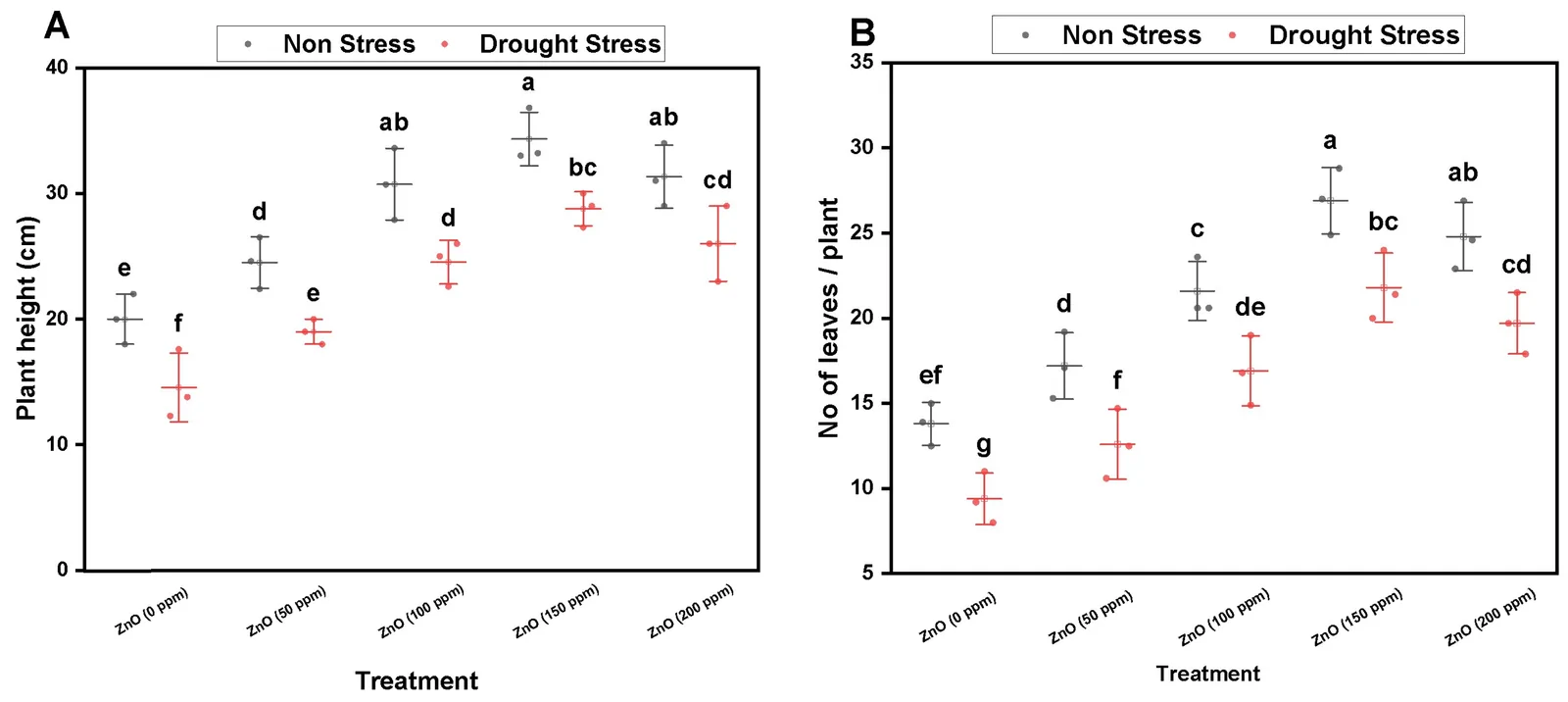
Effect of foliar-applied ZnO nanoparticles on (**A**) Plant height and (**B**) number of leaves/plant under non-stress and drought-stress conditions. Error bars represent the standard deviation of three replicates (*n* = 3). Different lowercase letters indicate significant differences among treatments according to the LSD test at *p* < 0.05.

Drought stress and ZnO nanoparticle treatments significantly affected plant growth attributes (Figure 2). Drought stress significantly reduced plant height and the number of leaves per plant compared to the non-stress condition. However, foliar application of ZnO nanoparticles significantly relieved the adverse effects of drought and induced vegetative growth. The maximum plant height was obtained at 150 ppm ZnO (34.33 cm), which is statistically higher than the untreated control (20.00 cm) under well-watered conditions. A similar trend was observed under drought stress, where 150 ppm ZnO produced the maximum plant height (28.77 cm), significantly higher than the drought-stressed control (14.57 cm) (Figure 2A). Similarly, drought stress significantly reduced the number of leaves per plant, while applying ZnO nanoparticles to the leaves significantly increased leaf production. Under both well-watered (26.90 leaves/plant) and drought-stressed (21.80 leaves/plant) conditions, the highest number of leaves occurred at 150 ppm ZnO, well above the respective untreated controls (Figure 2B).

### 2.3. Measurement of Fresh Leaf Weight, Root Weight and Total Pot Weight

**Figure 3:**
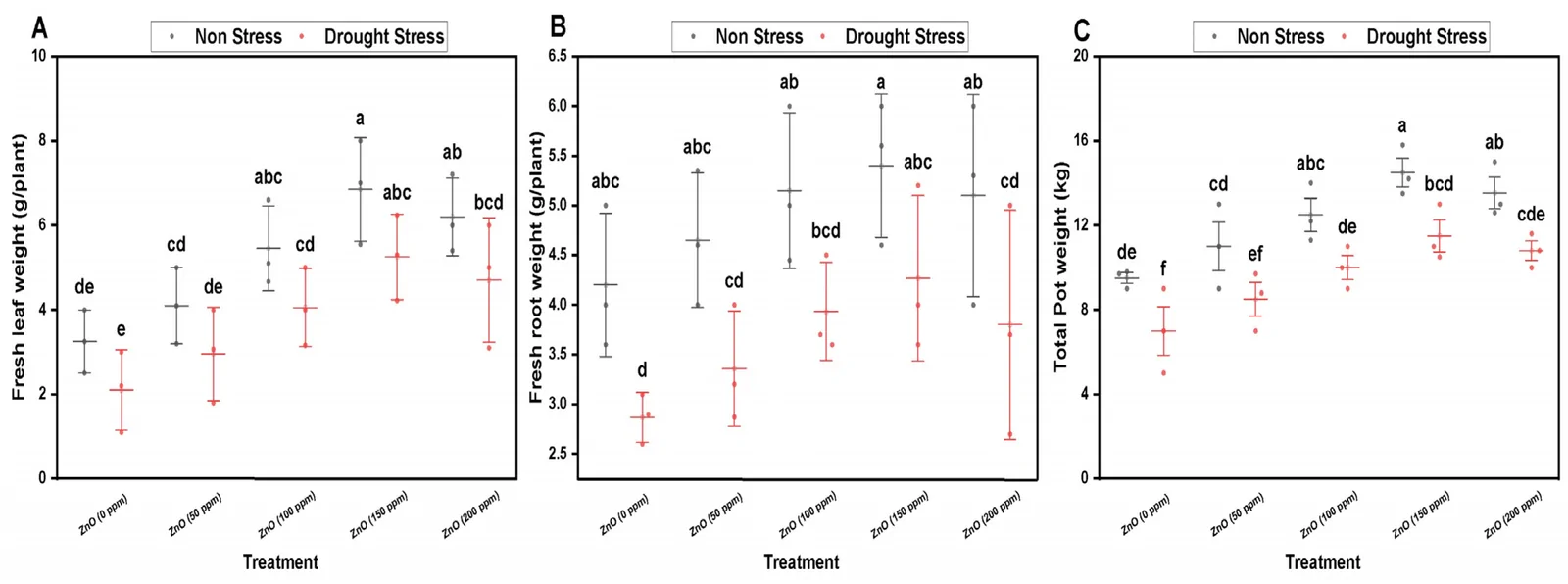
Effect of foliar-applied ZnO nanoparticles on fresh leaf weight (**A**), fresh root weight (**B**), and total pot weight (**C**) in citrus seedlings grown under non-stress and drought stress conditions. Error bars represent the standard deviation of three replicates (*n* = 3). Different lowercase letters above the bars represent significant differences between treatments according to the LSD test at *p* < 0.05.

The fresh biomass attributes of citrus seedlings were greatly influenced by drought stress and ZnO nanoparticle application, as shown in Figure 3. Drought stress significantly reduced fresh leaf and root weight, as well as total pot weight, compared with the non-stress condition. However, foliar application of ZnO nanoparticles significantly improved the adverse effects of drought stress and increased biomass accumulation. Fresh leaf weight increased significantly under non-stress (6.85 g/plant) and drought stress (5.25 g/plant) conditions at 150 ppm ZnO, compared with the untreated control (3.25 g/plant and 2.10 g/plant, respectively) (Figure 3A). Similarly, the 150 ppm ZnO treatment produced the highest fresh root weight under non-stress (5.40 g) and drought stress (4.27 g/plant) conditions, significantly higher than the untreated control (Figure 3B). Similarly, 150 ppm ZnO produced the highest total pot weight under both non-stress (14.50 kg) and drought stress (11.50 kg) conditions, significantly higher than the respective untreated controls (9.50 and 7.00 kg, respectively) (Figure 3C).

### 2.4. Measurement of Leaf Gas Exchange and Photosynthetic Attributes

**Figure 4:**
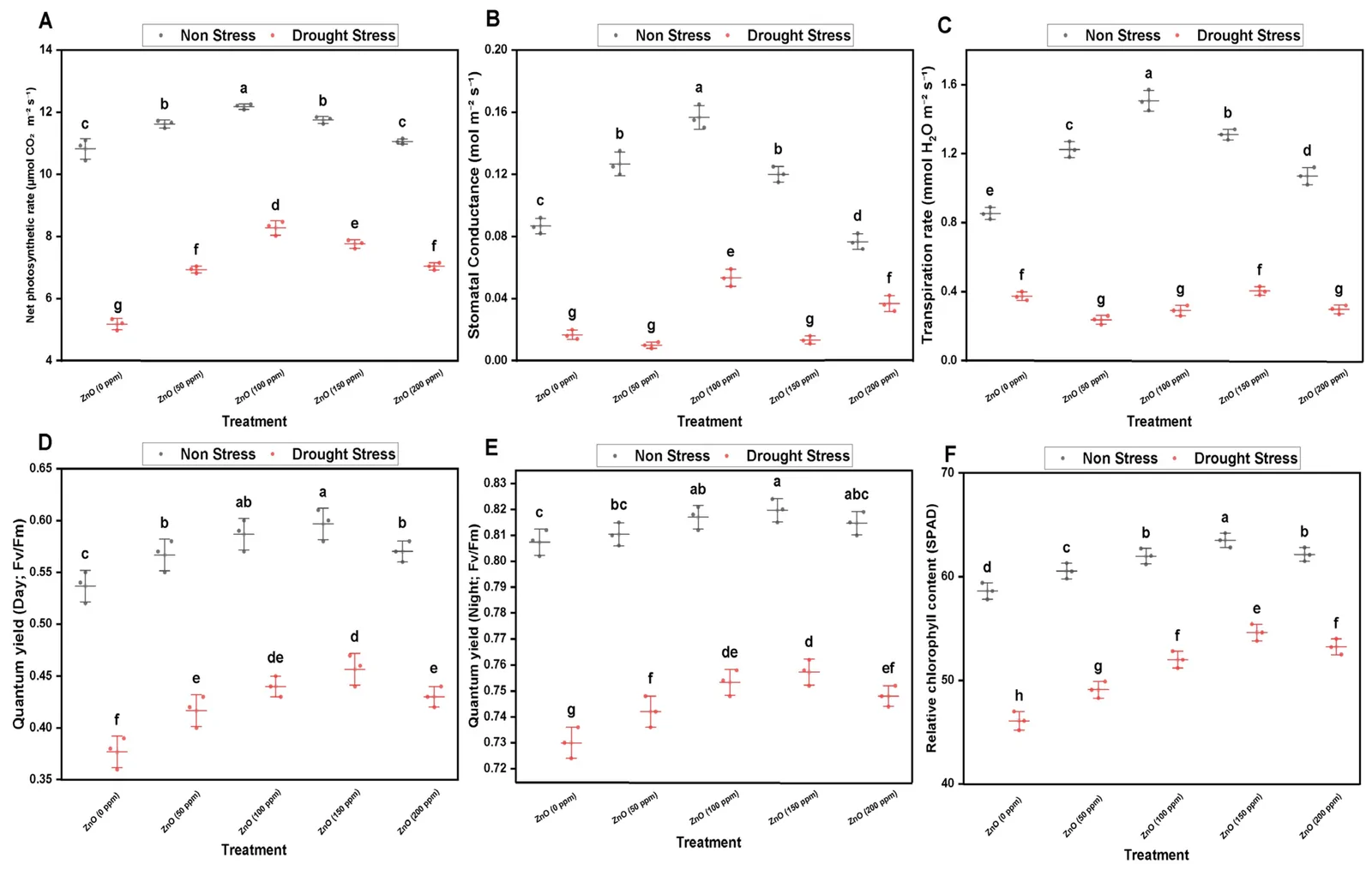
Effect of foliar-applied ZnO nanoparticles on net photosynthetic rate (Pn) (**A**), stomatal conductance (Gs) (**B**), transpiration rate (**C**), quantum yield of PSII during daytime (**D**), quantum yield of PSII during nighttime (**E**), and relative chlorophyll content (SPAD) (**F**) in citrus seedlings grown under non-stress and drought stress conditions. Error bars represent the standard deviation of three replicates (*n* = 3). Different lowercase letters above the bars represent significant differences between treatments according to the LSD test at *p* < 0.05.

Drought stress and ZnO treatments significantly affected leaf gas exchange and photosynthetic attributes of citrus seedlings (Figure 4). Under drought stress, net photosynthetic rate (Pn), stomatal conductance (Gs), transpiration rate (E), quantum yield of PSII during both day and night, and relative chlorophyll content (SPAD) decreased compared with the non-stress condition. Foliar application of ZnO enhanced these parameters under both irrigation treatments. Under non-stress and drought conditions, the highest Pn and Gs were recorded at the respective effective ZnO concentrations (12.18 µmol CO_2_ m^−2^ s^−1^ and 0.156 mol m^−2^ s^−1^), which were higher than untreated controls (8.27 µmol CO_2_ m^−2^ s^−1^ and 0.053 mol m^−2^ s^−1^) (Figure 4A,B). The highest E value under non-stress conditions was at 100 ppm ZnO (1.51 mmol H_2_O m^−2^ s^−1^), and ZnO application increased E under drought stress more than in the control (Figure 4C). PSII quantum yield was highest under 150 ppm ZnO under both irrigation regimes and during both day and night, compared with the corresponding controls (Figure 4D,E). The highest SPAD was obtained at 200 ppm ZnO, both in the absence of stress (63.5) and under drought stress (54.6), and was significantly higher than the respective controls (Figure 4F).

### 2.5. Measurement of Antioxidant Enzymes and DPPH Radical-Scavenging Activities

**Figure 5:**
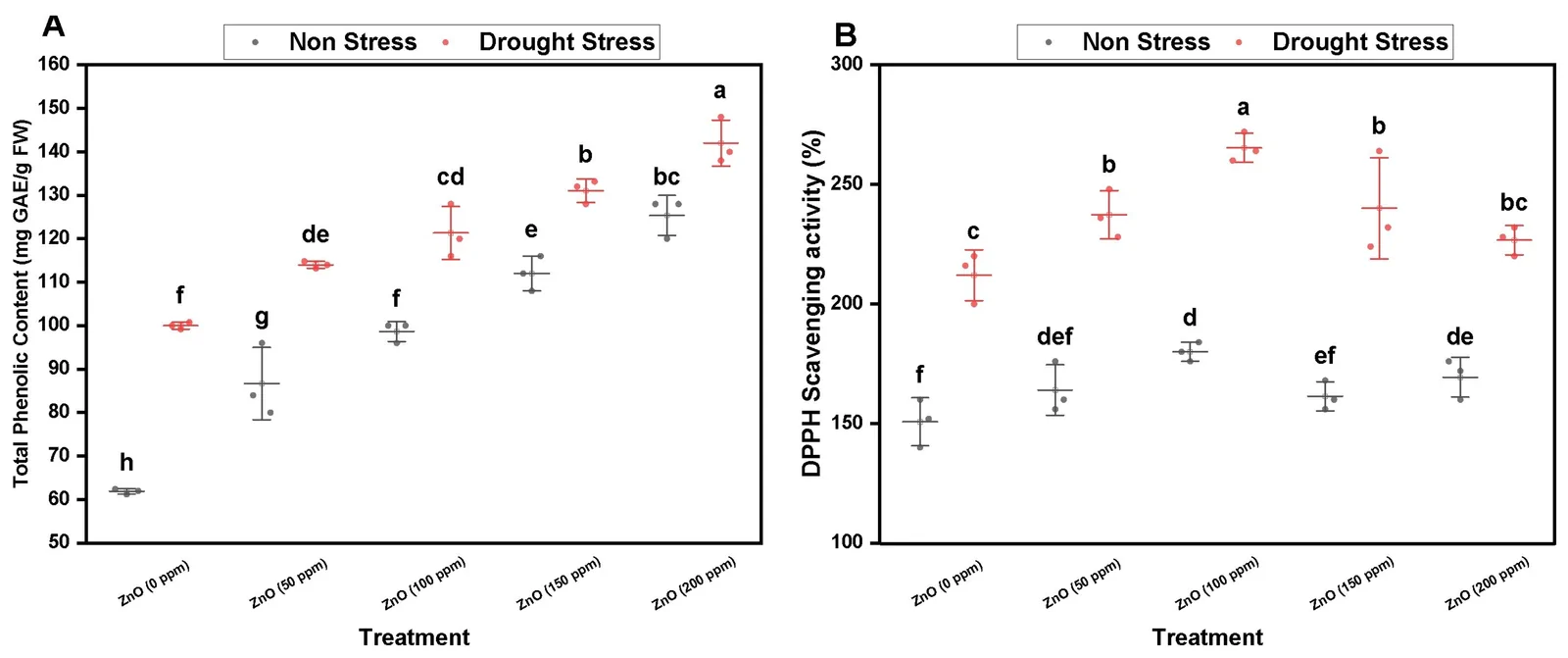
Effect of foliar-applied ZnO nanoparticles on total phenolic content (**A**) and DPPH scavenging activity (**B**) in citrus seedlings grown under non-stress and drought-stress conditions. Error bars represent the standard deviation of three replicates (*n* = 3). Different lowercase letters above the bars represent significant differences between treatments according to the LSD test at *p* < 0.05.

Drought stress and ZnO treatments significantly modulated total phenolic content (TPC) and DPPH radical-scavenging activity (Figure 5). Under non-stress conditions, TPC increased with ZnO concentration, reaching a maximum of 125.33 mg GAE/g FW at 200 ppm, followed by 112.00 and 98.67 mg GAE/g FW at 150 and 100 ppm ZnO, respectively, compared with 61.87 mg GAE/g FW in the untreated sample. The same trend was observed under drought stress, but with even higher TPC at 200 ppm ZnO (142.00 mg GAE/g FW), 131.07 mg GAE/g FW at 150 ppm, and 121.33 mg GAE/g FW at 100 ppm (Figure 5A). Under drought stress, DPPH radical-scavenging activity also increased; the highest values were obtained at 100 ppm ZnO (265.33%), 150 ppm ZnO (240.00%), and 50 ppm ZnO (237.33%), whereas it was lowest in the non-stressed control (150.67%). At 100 ppm ZnO, the highest DPPH activity (180.00%) was obtained, which was better than the untreated control (Figure 5B) under non-stress conditions. The results showed that ZnO application increased phenolic content and radical-scavenging activity more effectively under drought than under control conditions.

### 2.6. Measurement of Activity of Superoxide Dismutase, Catalase, and Peroxidase Activities

**Figure 6:**
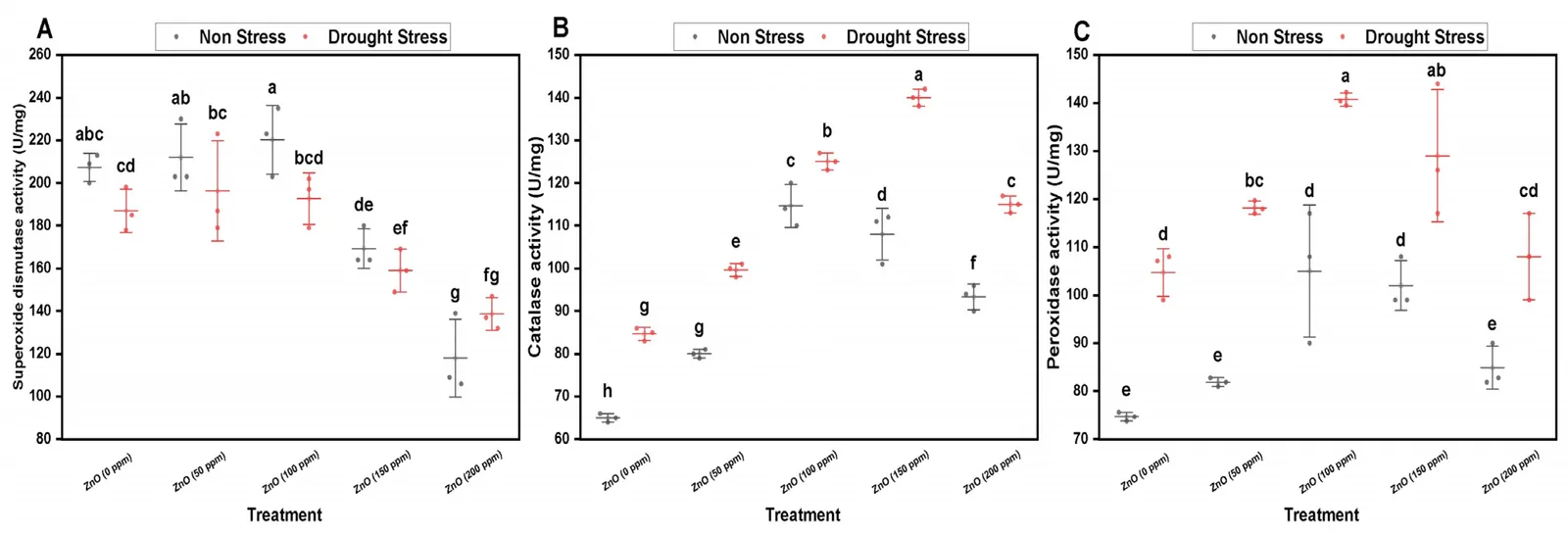
Effect of foliar-applied ZnO nanoparticles on superoxide dismutase activity (**A**), catalase activity (**B**), and peroxidase activity (**C**) in citrus seedlings grown under non-stress and drought stress conditions. Error bars represent the standard deviation of three replicates (*n* = 3). Different lowercase letters above the bars represent significant differences between treatments according to the LSD test at *p* < 0.05.

The activity of SOD, CAT, and POD was significantly influenced by drought stress and ZnO treatments (Figure 6). Under non-stress conditions, 100 ppm ZnO recorded the highest SOD activity (220.33 U/mg). In contrast, under drought stress, the concentration of 100 ppm ZnO recorded the highest activity of SOD (192.67 U/mg) compared with other treatments and the untreated control (Figure 6A). The highest CAT activity was obtained at 100 ppm ZnO (114.67 U/mg) and 150 ppm ZnO (140.00 U/mg) compared with their respective untreated controls (65.00 and 84.67 U/mg, respectively) (Figure 6B). POD activity was significantly higher at 100 ppm ZnO under both non-stress (105.00 U/mg) and drought stress (140.70 U/mg) conditions than in the corresponding non-stressed (74.70 U/mg) and drought-stressed controls (104.70 U/mg) (Figure 6C).

### 2.7. Pearson Correlation Analysis

**Figure 7:**
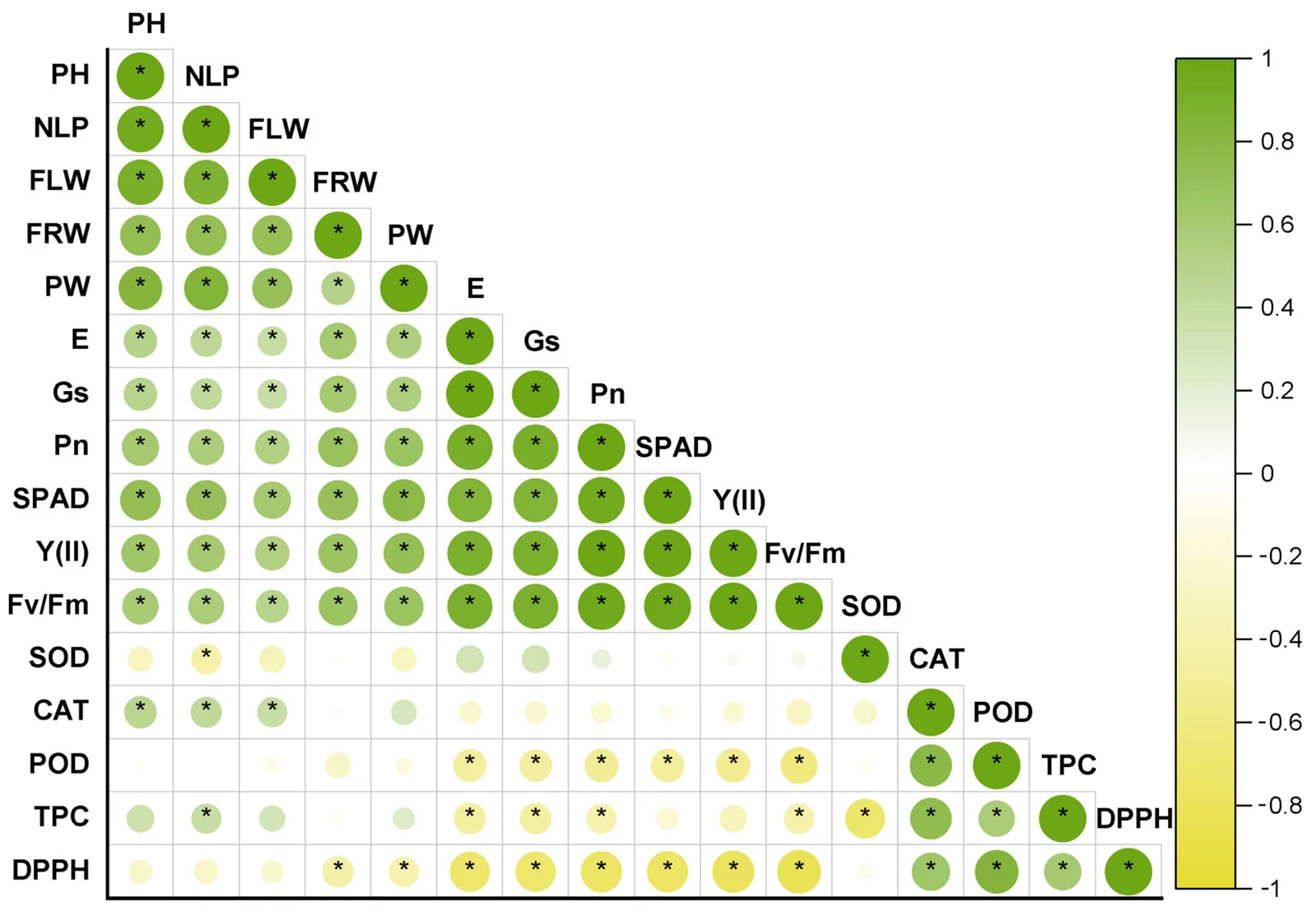
Pearson correlation heat map of the multidimensional relationships among morphological, physiological, and biochemical characteristics of rough lemon citrus rootstocks under different ZnO nanoparticle treatments and moisture levels. The correlation analysis was used to plot the two-way interdependence of the physical growth parameters (PH, NLP, FLW, FRW, PW), the gas exchange and photosynthetic parameters (E, Gs, Pn, SPAD, Y(II), Fv/Fm), and the biochemical oxidative stress indicators (SOD, CAT, POD, TPC, DPPH); * correlation is significant at the 0.01 level.

Pearson correlation analysis assessed relationships among growth, physiological, photosynthetic, biochemical, and antioxidant parameters in citrus seedlings exposed to different concentrations of ZnO nanoparticles under drought stress (Figure 7). The correlation heatmap revealed that most growth and photosynthetic parameters were significantly and positively correlated, indicating a close association among plant growth, gas exchange, chlorophyll content, and photosynthetic efficiency. In contrast, antioxidant-related biochemical parameters exhibited distinct correlation patterns with physiological and photosynthetic traits. Total phenolic content and DPPH radical-scavenging activity positively correlated with catalase and peroxidase activities, suggesting coordinated activation of the antioxidant defense system under drought stress. Several antioxidant parameters were negatively correlated with photosynthetic parameters, suggesting an inverse relationship between antioxidant defense responses and photosynthetic activity. The strong correlations among growth, photosynthetic, and antioxidant parameters indicated coordinated physiological and biochemical responses in citrus seedlings to nanoparticle application under different irrigation regimes. Treatment was positively correlated with plant growth, photosynthetic efficiency, and antioxidant defense, providing evidence of an antioxidant role in citrus seedlings that may contribute to increased drought resistance.

### 2.8. Principal Component Analysis (PCA)

**Figure 8:**
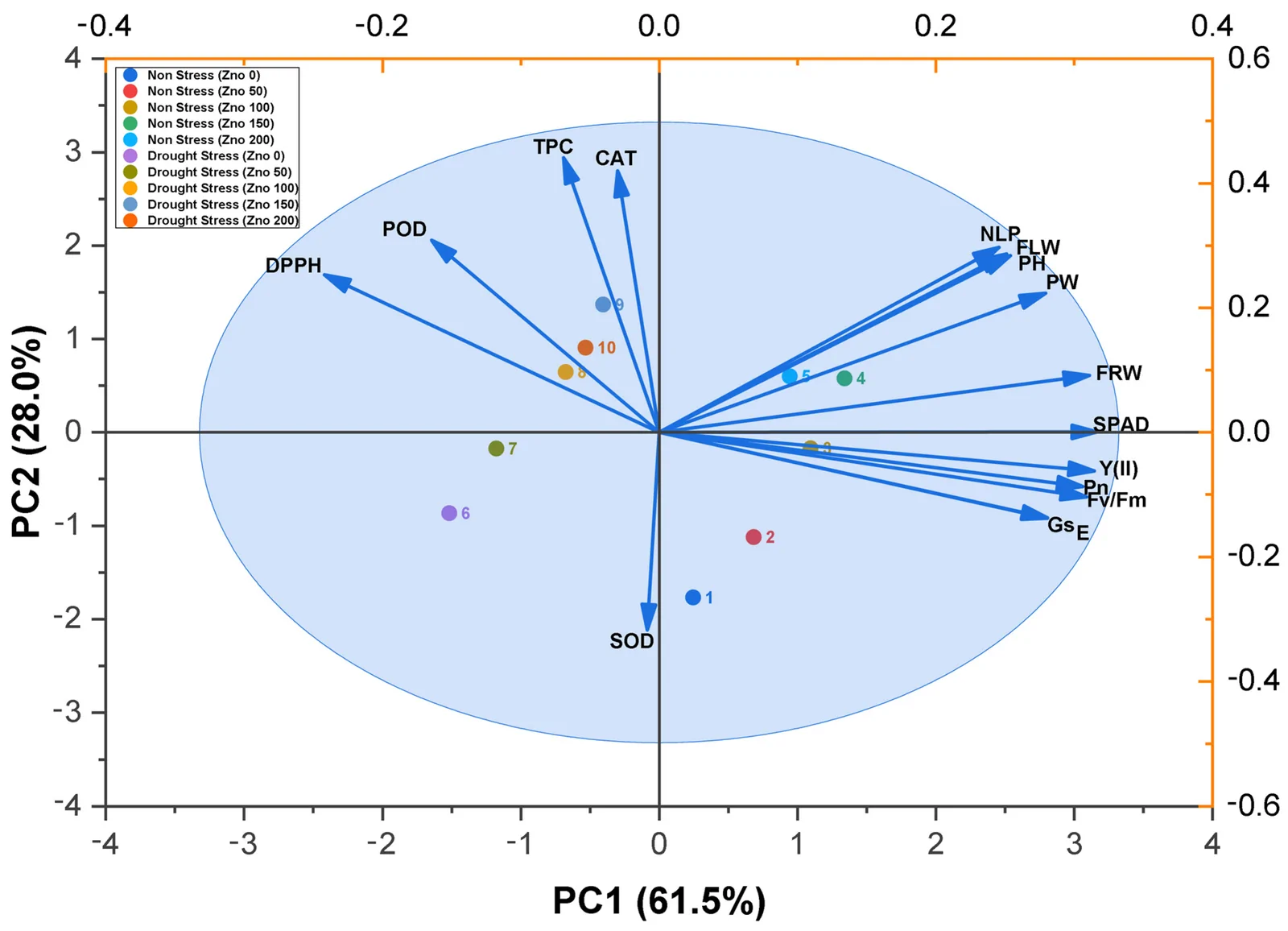
Principal Component Analysis (PCA) biplot showing the spatial segregation of experimental treatments and multidimensional trait associations in rough lemon citrus rootstocks. The dataset variance was effectively explained (89.5%) in the first two principal components, with PC1 (61.5%) and PC2 (28.0%) capturing 61.5% and 28.0%, respectively.

Principal Component Analysis (PCA) was performed to evaluate the multivariate relationships among growth, physiological, photosynthetic, biochemical, and antioxidant parameters under different ZnO nanoparticle treatments and irrigation regimes (Figure 8). The first two principal components explained 89.5% of the total variance, with PC1 and PC2 accounting for 61.5% and 28.0%, respectively. Most growth and photosynthesis-related parameters were positively associated with PC1, indicating a close relationship between plant growth, photosynthetic performance, and chlorophyll content. In contrast, antioxidant-related parameters were primarily associated with PC2, reflecting their contribution to the antioxidant defense system under drought stress. The PCA biplot clearly separated treatments by irrigation regime and ZnO nanoparticle concentration. Treatments under non-stress conditions were mainly distributed along the positive side of PC1. They were closely associated with improved growth and photosynthetic traits. In contrast, samples under water-deficit conditions were clearly separated from non-stress conditions and showed stronger associations with antioxidant-related parameters. Among ZnO treatments, moderate concentrations (100–150 ppm) were strongly correlated with improved vegetative growth, gas-exchange characteristics, and relative chlorophyll content, whereas higher antioxidant activities were correlated with drought-stress treatments. Overall, the PCA results revealed that ZnO nanoparticle supplementation significantly improved physiological performance and enhanced antioxidant defense, both of which are important for mitigating drought-induced stress in citrus seedlings.

## 3. Discussion

Drought is a major environmental challenge to citrus growth and productivity, as water deficit reduces plant water status, limits stomatal conductance and photosynthetic carbon assimilation, and consequently reduces biomass production. The present study showed that water limitation negatively affected vegetative growth in rough lemon seedlings by significantly reducing plant height, leaf number, fresh leaf weight, and fresh root weight. Limited water availability reduced metabolic activity and carbon assimilation, leading to similar reductions in growth in other crops (e.g., maize, tomato, and soybean) under drought conditions [27,28]. Water deficits can adversely affect vegetative growth by affecting cell division and cell expansion, stomatal conductance, and photosynthetic carbon assimilation [29,30]. In the current study, NPs-treated lemon seedlings showed significant growth inhibition under drought stress. In contrast, foliar application of green-synthesized ZnO nanoparticles effectively mitigated these adverse effects. It promoted vegetative growth, particularly at the optimal concentration (100–150 ppm), demonstrating their beneficial role in improving drought tolerance.

We evaluated the formation and physicochemical characteristics of the green-synthesized ZnO nanoparticles using complementary spectroscopic, crystallographic, and microscopic approaches (Figure 1). The ZnO nanoparticle extract showed an optical absorption profile different from the *C. gigantea* leaf extract in the UV–Visible spectrum (Figure 1A), suggesting that ZnO developed an optical response after synthesis. Such alterations in the UV–Visible absorption profiles have been reported in the synthesis of ZnO nanoparticles in the presence of plants and are a primary criterion used to confirm the formation of nanoparticles [31,32].

The crystalline nature and phase identity of the synthesized material were further confirmed by XRD analysis (Figure 1B), which showed diffraction reflections of the hexagonal wurtzite structure of ZnO that matched JCPDS No. 36-1451, as previously reported for phytogenic ZnO nanoparticles [33].

No other large diffraction features were observed under the conditions analyzed, indicating that no large impurity crystalline phase was detected. The SEM analysis (Figure 1C) showed that the morphology of the particles was mostly rounded/spherical with noticeable aggregation, which is a characteristic feature of biologically synthesized metal oxide nanoparticles [34,35]. DLS analysis also showed an average hydrodynamic diameter of around 10 nm (Figure 1D), with most particles within the nanoscale range. *C. gigantea* has been reported to possess a variety of secondary metabolites, including flavonoids, phenolics, alkaloids, tannins, and other bioactive substances that could participate in reduction, nucleation, surface capping, and stabilization during nanoparticle formation [36]. Previous studies using *C. gigantea* extracts have also reported crystalline products with nanoscale dimensions and defined morphologies, supporting this plant’s suitability as a botanical source for their synthesis. However, dedicated phytochemical and surface-chemical characterization is needed to identify the specific compounds responsible for these processes [37,38]. Overall, the UV–Visible, XRD, SEM, and DLS results provide complementary evidence for the formation of crystalline, spherical nanoparticles via the *C. gigantea*-mediated green synthesis approach.

The ability of biogenic ZnO nanoparticles to restore plant height and, most importantly, to generate the maximum number of leaves, particularly at levels below 150 ppm, is closely associated with the structural role of elemental zinc in stabilizing cell membranes during periods of high physiological distress. Zinc is an essential catalytic cofactor that regulates fundamental physiological processes, including activation of primary polymerases and mechanical stabilization of cell membrane architecture, thereby directly controlling continuous cellular division [39]. The nanoscale size of ZnO enables rapid absorption from the foliar surface and translocation within the plant, whereas conventional bulk zinc fertilizers have very low utilization efficiencies and become ineffective due to rapid soil immobilization [40]. Previous studies have suggested that foliar-applied ZnO nanoparticles are associated with improved photosynthetic performance and growth in rough lemon seedlings under drought stress.

Moreover, the recovery of fresh leaf weight (FLW) and fresh root weight (FRW) is notable, supporting efficient metabolic processes that depend on the accurate utilization of nanoscale micronutrients. Drought stress reduces soil water availability and movement, restricting nutrient uptake and root activity, thereby decreasing the acquisition of key macronutrients and increasing energy spent to maintain cellular homeostasis [41]. Foliar application of biogenic ZnO NPs improved plant water status and supported biomass production under drought stress [42]. At 150 ppm ZnO, the macroscopic biomass is protected against the degrading effects of stress, indicating that optimized botanical nanotechnology can adequately preserve the physical yield components of fragile citrus rootstocks.

One of the most basic physiological costs of an extended drought is the systemic impairment of normal gas exchange, primarily due to stomatal closure in response to drought stress. Although stomatal resistance is an important survival strategy to avert fatal desiccation, it simultaneously inhibits carbon dioxide uptake, thereby suppressing the net photosynthetic rate (Pn) and further reducing photosynthetic efficiency [43,44]. Experimental evidence indicates that foliar application of 100 ppm biogenic zinc effectively opens stomatal pores, thereby restoring a functional balance between the necessary CO_2_ uptake and the loss of transpiration water vapor [45,46].

Under drought stress, reduced stomatal conductance (Gs) limits CO_2_ availability for photosynthesis, while prolonged water deficit impairs chlorophyll content and photosystem II (PSII) efficiency, thereby reducing photosynthetic performance [47,48]. In the present study, foliar application of 150 ppm ZnO nanoparticles maintained the highest relative chlorophyll content (SPAD) and the highest daytime and nighttime PSII quantum yields under drought stress, indicating improved preservation of photosynthetic performance. Since zinc plays an important role in chlorophyll biosynthesis, highly bioavailable nano-zinc is useful for protecting foliar tissues against excessive photo-oxidative bleaching and premature chlorosis [49,50].

The observed improvements in photosynthetic performance and plant growth may be associated with the physicochemical characteristics of the synthesized ZnO nanoparticles. However, the present study did not directly examine the underlying mechanisms. The use of traditional micronutrients often fails to reduce damage from terminal drought, since large molecular compounds cannot penetrate the waxy cuticular surfaces of the most severely stressed foliage [51,52]. On the other hand, the large surface-area-to-volume ratio of ZnO nanoparticles enables their rapid cellular uptake, supplying active zinc ions to damaged chloroplast machinery [53]. Compared with conventional zinc fertilizers, ZnO nanoparticles deliver zinc more efficiently, thereby supporting photosynthetic performance under drought stress.

Alterations in cellular homeostasis associated with prolonged water deficit are invariably accompanied by severe oxidative stress, characterized by the accumulation of reactive oxygen species, including hydrogen peroxide and superoxide anions. These are highly reactive oxygen intermediates that aggressively attack primary cellular structures, triggering massive lipid peroxidation cascades that critically impair the structure and selective permeability of lipid membranes [54,55]. Under oxidative stress, plants dynamically upregulate a complex network of intrinsic defense responses that depend heavily on enzymatic networks and non-enzymatic scavengers to neutralize reactive intermediates before they cause irreversible cellular senescence [56]. Although untreated drought-stressed citrus rootstocks in this experiment showed naturally elevated defense markers, this unassisted response was ineffective at halting the macroscopic degradation of vegetative development.

Applying biogenically synthesized ZnO nanoparticles significantly enhanced antioxidant enzyme activities in rough lemon seedlings under drought stress. Treatment with 100–150 ppm ZnO increased catalase (CAT) and peroxidase (POD) activities, which play important roles in the detoxification of hydrogen peroxide into water and oxygen [57]. Enhanced antioxidant enzyme activities following ZnO nanoparticle application may contribute to improved protection against oxidative stress, thereby helping to maintain photosynthetic function under drought conditions [58,59]. These findings suggest that ZnO nanoparticles enhanced the antioxidant defense system, which may have contributed to the improved photosynthetic performance observed under drought stress.

At the same time, biogenic ZnO nanoparticles significantly enhanced non-enzymatic antioxidant capacity, resulting in a dose-dependent increase in total phenolic content (TPC) and DPPH radical-scavenging activity. Phenolic compounds are important non-enzymatic antioxidants that contribute to scavenging reactive oxygen species and reducing oxidative damage under drought stress [60,61]. The concurrent increase in TPC, together with antioxidant enzyme activities, suggests that ZnO nanoparticles enhanced the overall antioxidant defense system, which may have contributed to improved drought tolerance in rough lemon seedlings [62].

Traditionally used zinc sulfate fertilizers tend to have poor nutrient-use efficiency, as significant amounts of zinc are immobilized in soil or leached [63]. Moreover, drought stress causes soil moisture loss, which limits the transport of nutrients from the rhizosphere to the plant surface and thus hinders the uptake of nutrients, thereby influencing plant growth and physiological response [64,65]. In such conditions, the use of conventional fertilizers can further decrease nutrient efficiency and pose environmental risk due to excessive fertilizer inputs [66,67].

ZnO nanoparticles can be used as a potential strategy to enhance nutrient delivery under drought stress conditions. The nanoparticles are extremely small, allowing them to be applied to the leaves and provide a high surface area-to-leaf-volume ratio, which would increase the availability of zinc to leaf tissues and facilitate other physiological processes related to plant growth and photosynthesis [68,69]. Some previous studies have indicated that foliar application of ZnO nanoparticles can facilitate the uptake of nutrients and physiological growth under water deficit conditions [70]. The results are also consistent with the present study, where plant growth, photosynthetic activity, and antioxidant enzyme activities increased with ZnO nanoparticle application under drought stress. The same has also been reported by [71], who showed that the use of nano-fertilizers led to higher efficiency in nutrient use and drought tolerance in plants.

The use of ZnO nanoparticles in the agricultural field is very promising, but traditional chemical and physical methods of synthesis are often high-energy, high-temperature, and involve chemical reactants that can generate undesirable by-products for the environment [72]. Furthermore, some chemical synthesis methods rely on stabilizing agents that can leave behind traces of chemicals on the surfaces of nanoparticles that can alter their interactions with biological systems [73]. Microorganisms have also been studied for biosynthesis, but large-scale production is often limited by the requirement for aseptic culture and downstream processing of a relatively complex nature [74].

In the present study, foliar application of *C. gigantea*-mediated ZnO nanoparticles correlated with better photosynthetic performance, growth, and antioxidant responses in rough lemon seedlings under drought. The reported presence of *C. gigantea* in arid and saline environments like the Thar Desert, along with its saline tolerance, implies potential for using marginal land for cultivation [75,76]. However, biomass productivity, cultivation costs, and the sustainability of large-scale biomass supply need evaluation. The presence of various reported secondary metabolites, such as flavonoids and alkaloids [77,78], may help form and stabilize nanoparticles [79]. Several limitations should be considered when interpreting these findings: lipid peroxidation (malondialdehyde), tissue zinc concentrations, and zinc uptake and transport were not determined, precluding mechanistic interpretation. Enhancing antioxidant activities alone, without demonstrating reduced oxidative damage, was insufficient, and the mechanism behind the observed responses related to enhanced zinc delivery could not be confirmed. However, we did not assess synthesis yield, production scalability, cost, or environmental safety. Future studies should address these mechanistic gaps using targeted biochemical measurements and zinc analyses, along with molecular approaches, phytochemical profiling, and further characterization of particle-size distribution, surface charge, elemental composition, surface chemistry, and colloidal stability. Practical feasibility should be evaluated through quantitative production assessments at different scales, based on biomass input, resources used, and processing costs, as well as life cycle assessment and ecotoxicological testing. Additionally, longer-term experiments and field trials with other citrus genotypes and scion–rootstock combinations are needed to determine whether the responses observed in this 15-day seedling experiment scale to other citrus production systems.

## 4. Materials and Methods

### 4.1. Experimental Site and Microclimate Methods

An in vivo pot experiment was conducted systematically in the controlled glasshouse nursery at the Department of Horticulture, Bahauddin Zakaria University, Multan, Pakistan (Latitude 30.2570° N; Longitude 71.5150° E). A tightly controlled microclimate was essential to isolate physiological and biochemical changes in citrus rootstock in response to drought stress and eliminate confounding environmental factors, such as unpredictable rainfall or extreme weather fluctuations. The inside of the glasshouse was carefully monitored throughout the experiment, which began at the end of February 2023. The average daytime temperature was 34 °C, and nighttime temperatures were maintained at around 23 °C. To simulate a realistic semi-arid climate that increases transpiration pull, relative humidity was kept low, averaging 26 percent under natural sunlight.

The biological sample used for the research was 1-year-old rough lemon (*Citrus jambhiri* L.) rootstock. Thirty uniform seedlings of the same size and type, with canopy, stem girth, and root development, were obtained from the certified nursery of the Department of Horticulture, Bahauddin Zakariya University, Multan, Pakistan. Seedlings were pre-established and one year old, in standardized clay pots (30 cm diameter) with sandy loam soil. The original nursery soil was left in place, and no further soil drying, sieving, or autoclaving treatment was undertaken before the experiment.

### 4.2. Plant Material Selection and Preparation

Botanical samples of the family Apocynaceae, locally known as the Aak plant (*Calotropis gigantea*), were obtained from the flora reserves of Bahauddin Zakaria University. The fully developed green leaves were carefully collected and transported for the preparation of ZnO nanoparticles. The samples were washed several times with sterile distilled water to remove surface dust and atmospheric particulates. After sanitization, the leaves were sun-dried to evaporate any remaining internal moisture and preserve the primary capping agents for subsequent bioreduction. The dried leaves were then prepared for extraction and subsequent use in the green synthesis of ZnO nanoparticles under the experimental conditions described below.

### 4.3. Biogenic Synthesis Protocol for ZnO Nanoparticles

**Figure 9:**
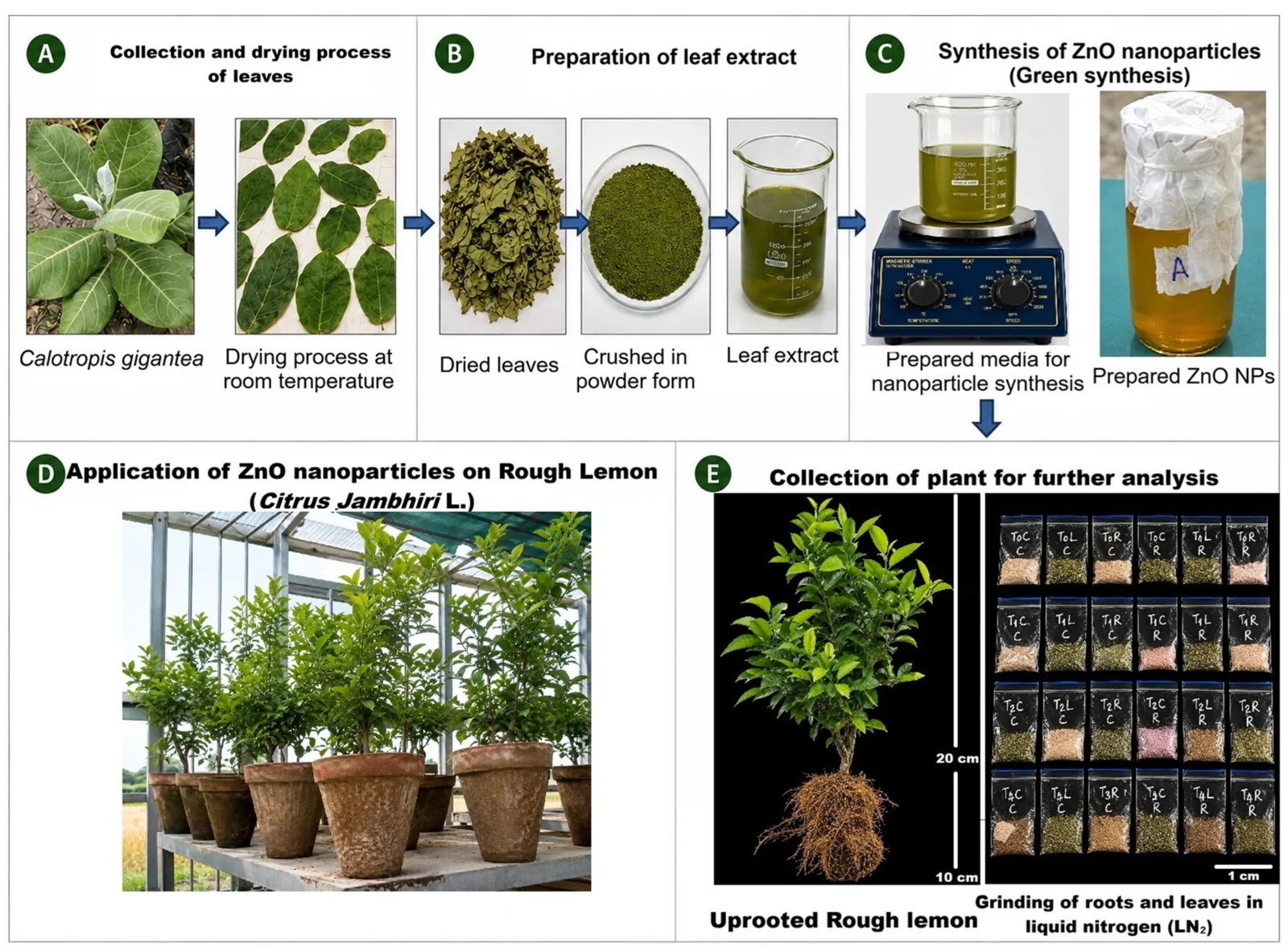
Schematic workflow of *C. gigantea*-mediated ZnO nanoparticle synthesis and its application to rough lemon (*Citrus jambhiri* L.). (**A**) Collection and room-temperature drying of *C. gigantea* leaves; (**B**) preparation of *C. gigantea* leaf extract; (**C**) green synthesis and preparation of ZnO nanoparticles; (**D**) foliar application of ZnO nanoparticles to rough lemon plants under greenhouse conditions; and (**E**) uprooting of rough lemon plants and collection of root and leaf tissues for subsequent analyses.

A highly concentrated aqueous extract was prepared by adding 50 g of washed, dried, and finely macerated *C. gigantea* leaves to a glass beaker containing 100 mL of sterile distilled water (Figure 9A). The botanical suspension was boiled continuously for 60 min until the aqueous solution turned light yellow, indicating that the phytochemical reducing agents had been successfully solubilized. The resulting broth was cooled, vacuum-filtered through high-grade Whatman filter paper (Cytiva, Maidstone, UK), and refrigerated (Figure 9B). The same plant-extract preparation techniques used to prepare green nanoparticles have been reported in prior studies [80,81].

For the green synthesis of zinc oxide nanoparticles (Figure 9C), 50 mL of the prepared leaf extract was placed on a hot plate with a magnetic stirrer (DAIHAN Scientific, Wonju, Republic of Korea) and heated continuously to 60–80 °C. After the internal temperature had reached 60 °C, 5 g of high-purity zinc nitrate (Sigma-Aldrich, St. Louis, MO, USA) was added to the agitating solution. The mixture was stirred and heated until all the liquid was driven off, forming a very thick, deep-yellow paste. The paste was poured into a ceramic crucible and calcined at a high temperature in an air-fired laboratory furnace (Nabertherm GmbH, Lilienthal, Germany), with every effort to maintain a temperature of 400 °C throughout the process (2 h). We allowed the resulting light-yellow solid to cool, then manually pulverized it in a mortar and pestle to obtain an ultra-fine, homogeneous nanoparticle powder. This ZnO nanoparticle was synthesized using the plant-mediated synthesis and calcination method, which is commonly used in recent studies on nanobiotechnology [82,83]. We prepared a 1000 ppm ZnO NP stock suspension by dispersing 1 g of the synthesized powder in 1000 mL of distilled water. We selected concentrations of 50, 100, 150, and 200 ppm based on previously reported effective and non-phytotoxic application ranges for citrus crops, along with preliminary laboratory screening results. We then diluted appropriate volumes of the stock suspension with distilled water to obtain the required foliar application concentrations (Figure 9D). On Day 15, the plants were carefully uprooted, and leaf and root tissues were collected for subsequent physiological and biochemical analyses (Figure 9E); the detailed experimental timeline, including drought imposition on Day 0 and the initiation of foliar ZnO nanoparticle application on Days 3–4, followed by spraying on alternate days, is described in Section 4.4.

### 4.4. Nanomaterial Synthesis and Drought Imposition

**Figure 10:**
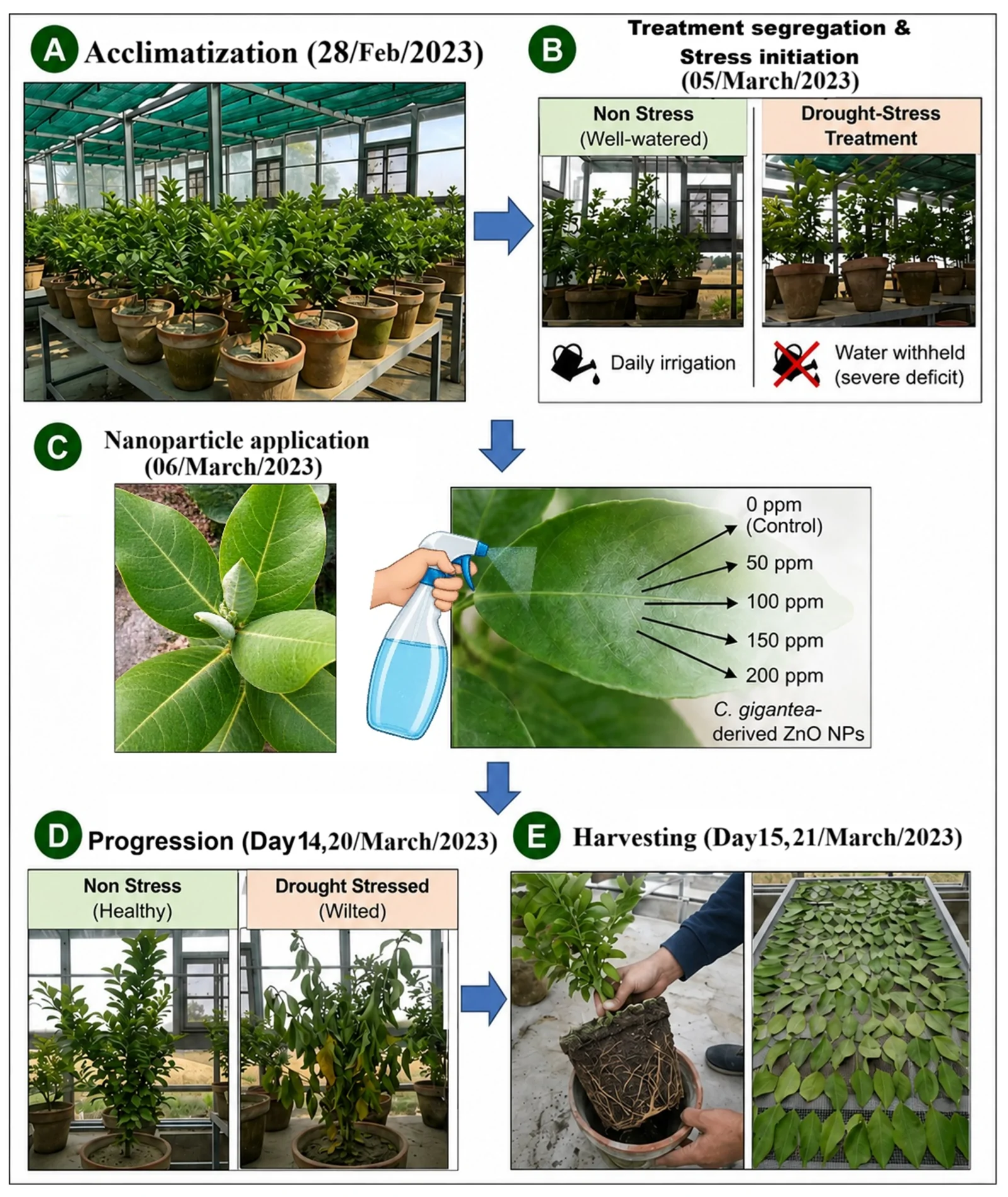
Experimental timeline showing (**A**) acclimatization of rough lemon seedlings at field capacity before Day 0; (**B**) drought imposition by withholding irrigation on Day 0; (**C**) the first foliar application of *C. gigantea*-mediated ZnO NPs at 0, 50, 100, 150, and 200 ppm on Days 3–4, followed by applications on alternate days; (**D**) plant responses on Day 15; and (**E**) final harvesting and tissue collection on Day 15.

The in vivo evaluation of the synthesized ZnO nanoparticles was performed using a completely randomized design (CRD) with two factors in a factorial arrangement. Thirty uniform, one-year-old rough lemon (*Citrus jambhiri* L.) seedlings were grown under field capacity till acclimatization was carried out on 28 February 2023 (Figure 10A). The seedlings were then divided into two irrigation treatments: well-watered (watered daily to keep the field moist) and drought-stressed (irrigation was withheld). Day 0 of drought treatment was chosen on this date (Figure 10B). For both irrigation regimes, plants received foliar applications of ZnO nanoparticles (0, 50, 100, 150, and 200 ppm), with three replications per treatment. The first foliar application was carried out on 6 March 2023, and the subsequent applications were done on alternate days until the end of the experiment (Figure 10C). Plants under well water were watered regularly to maintain adequate soil moisture and plant turgidity, while plants under drought stress were not watered during the treatment period. As the drought progressed, stressed plants displayed water stress-related symptoms, such as leaf wilting, downturned leaves, and early dieback of the terminal shoots (Figure 10D). We observed the plants throughout the experimental period and harvested the leaves and roots on 21 March 2023 for physiological and biochemical analysis (Figure 10E).

### 4.5. Assessment of Plant Height and Number of Leaves per Plant

We took baseline measurements before the drought started and compared them with the last measurements taken before harvest. We measured total plant width with a standard flexible tape and measured the distance from the absolute soil surface in the vertical plane to the lowest viable shoot tip in centimetres. At the same time, per-plant counts of photosynthetically active leaves provided a proxy for canopy turgor maintenance and resistance to stress-induced abscission, providing a direct measure of the canopy’s ability to sustain cellular turgor and avoid stress-induced foliar abscission [84].

### 4.6. Measurement of Fresh Leaf Weight, Root Weight and Total Pot Weight

After non-destructive measurements, we performed a systematic destructive harvesting procedure to accurately partition biological mass between roots and shoots. First, we recorded the weight of the intact pot. We then carefully removed the rootstocks from the soil matrix and separated them into root and shoot portions. To remove residual clay and soil particles, we washed these fractions thoroughly with running tap water and shade-dried them to remove surface moisture. The weight of fresh leaves and fresh roots was then measured in (g) using a highly calibrated digital electronic balance (Mettler Toledo, Greifensee, Switzerland) [85,86].

### 4.7. Measurement of Leaf Gas Exchange and Photosynthetic Attributes

Leaf gas exchange parameters were measured to evaluate the photosynthetic efficiency of citrus rootstocks under drought and ZnO nanoparticle-induced water stress. The net photosynthetic rate (Pn), stomatal conductance (Gs), and transpiration rate (E) were recorded on fully expanded leaves on Days 5, 10, and 15 after the initiation of drought stress, between 08:00–10:00 a.m. under natural light using a portable photosynthesis system (LCi-SD, ADC BioScientific Ltd., Hoddesdon, UK) equipped with an infrared gas analyzer (IRGA).

The maximum quantum efficiency of Photosystem II (PSII) (Fv/Fm) was determined after dark adaptation for 25–30 min using a FluorPen FP-100 portable chlorophyll fluorometer (Photon Systems Instruments, Drásov, Czech Republic) [87].

The lowest fluorescence (Fo) and highest fluorescence (Fm) were measured after a saturating light pulse, and the maximum quantum yield of PSII was determined as Fv/Fm = (Fm − Fo)/Fm [88]. We conducted measurements during the day and at night to evaluate PSII photochemical performance under well-watered and drought-stress conditions [89]. Moreover, the relative chlorophyll content was also measured by applying a SPAD-502 chlorophyll meter (Konica Minolta, Tokyo, Japan) to expanded leaves as a non-destructive, quick measure of the leaf chlorophyll concentration [90,91].

### 4.8. Measurement of Antioxidant Enzymes and DPPH Radical-Scavenging Activity

We assayed harvested tissues to quantify moisture-deficit-induced biochemical degradation and the associated defense response in biogenic nanoparticles. Immediately after harvest, we flash-froze isolated leaf and root samples in liquid nitrogen to prevent enzymatic degradation and maintain cellular homeostasis. We then prepared the finely crushed, frozen tissue homogenates for spectrophotometric analysis using a UV-1800 UV–Vis spectrophotometer (Shimadzu Corporation, Kyoto, Japan). This total capacity of the tissue to neutralize volatile oxygen intermediates was measured as Total Phenolic Content (TPC) using Folin–Ciocalteu reagent (Sigma-Aldrich, St. Louis, MO, USA) [92] and as the percentage of total DPPH (2,2-diphenyl-1-picrylhydrazyl, Sigma-Aldrich, St. Louis, MO, USA) radical scavenging activity [93].

### 4.9. Effect of Superoxide Dismutase, Catalase and Peroxidase Activities

The activities of superoxide dismutase (SOD), catalase (CAT), and peroxidase (POD) were determined using enzyme extracts. SOD activity was determined according to Kunos et al. [94] using a xanthine oxidase-based assay (Sigma-Aldrich, St. Louis, MO, USA) following the manufacturer’s instructions; absorbance was monitored at 550 nm for 1 min at 10 s intervals using an ELx800 microplate reader (BioTek Instruments, Winooski, VT, USA). CAT activity was determined according to Khan et al. [95]. Using the microplate procedure, in which 20 µL of enzyme sample was mixed with 100 µL of 5 mM H_2_O_2_ (Merck KGaA, Darmstadt, Germany) and incubated at 37 °C for 5 min, followed by the addition of 130 µL of working reagent (Sigma-Aldrich, St. Louis, MO, USA) and incubation at 25 °C for 5 min; absorbance was subsequently measured at 490 nm using the ELx800 microplate reader (BioTek Instruments, Winooski, VT, USA). POD activity was determined according to Zheng et al. [96]. We used the corresponding peroxidase assay reagents (Sigma-Aldrich, St. Louis, MO, USA). Enzyme activities were calculated according to the respective methods and expressed as units per milligram of protein (U/mg). These parameters were used to profile the concentration at which nanoscale zinc acts as a bioavailable elicitor, stimulating the plant’s natural defense responses under environmental stress.

### 4.10. Statistical Analysis

All the gathered data were statistically tested using a two-factor Analysis of Variance (ANOVA) in OriginPro (v2024 SR1 10.1.0.178) [97]. We compared treatment-group means using the Least Significant Difference (LSD) test at the 5 percent level to determine significant differences between groups. We performed a post hoc power analysis in OriginPro 2024 separately for the non-stress and drought-stress conditions to confirm the experimental design’s ability to detect the observed treatment effects. The observed power exceeded 0.80 in both conditions, indicating adequate sensitivity to detect the observed treatment effects.

## 5. Conclusions

Rough lemon (*Citrus jambhiri* L.) rootstocks treated with green-synthesized ZnO nanoparticles using *C. gigantea* extract showed better drought-tolerance responses in growth performance, physiological attributes, and antioxidant defense mechanisms. Under drought stress, vegetative growth parameters such as plant height, leaf number, and fresh biomass accumulation increased significantly with ZnO nanoparticle application, especially at 100–150 ppm. Additionally, ZnO nanoparticles improved plant gas exchange characteristics, chlorophyll content, and PSII activity, thereby promoting physiological activity in these plants under water stress. Increased activities of antioxidant enzymes such as superoxide dismutase (SOD), catalase (CAT), and peroxidase (POD), along with improved total phenolic content (TPC) and DPPH radical-scavenging activity, indicated a stronger antioxidant response under drought stress due to the application of ZnO nanoparticles. The 150 ppm ZnO nanoparticles produced the best growth responses, and moderate concentrations yielded the best physiological and biochemical responses. The results suggest that green-synthesized ZnO nanoparticles could be a promising biostimulant to enhance drought tolerance in citrus rootstocks. Further investigations to directly measure reactive oxygen species and markers of oxidative damage such as hydrogen peroxide and malondialdehyde (MDA), and to provide detailed physicochemical characterization of ZnO nanoparticles are needed to elucidate the mechanisms of drought tolerance mediated by ZnO nanoparticles. To support the practical use of ZnO nanoparticles in sustainable citrus production systems, future studies should incorporate molecular-based approaches and field-level evaluations. The 150 ppm ZnO nanoparticle concentration in foliar application emerged as optimal treatment under the tested conditions, as it conferred the strongest growth responses and enhanced photosynthetic and antioxidant characteristics under drought stress.

## Data Availability

The original contributions developed in this study are incorporated in the article. Further questions can be addressed to the respective authors.
